# First Expert Elicitation of Knowledge on Possible Drivers of Observed Increasing Human Cases of Tick-Borne Encephalitis in Europe

**DOI:** 10.3390/v15030791

**Published:** 2023-03-20

**Authors:** Claude Saegerman, Marie-France Humblet, Marc Leandri, Gaëlle Gonzalez, Paul Heyman, Hein Sprong, Monique L’Hostis, Sara Moutailler, Sarah I. Bonnet, Nadia Haddad, Nathalie Boulanger, Stephen L. Leib, Thierry Hoch, Etienne Thiry, Laure Bournez, Jana Kerlik, Aurélie Velay, Solveig Jore, Elsa Jourdain, Emmanuelle Gilot-Fromont, Katharina Brugger, Julia Geller, Marie Studahl, Nataša Knap, Tatjana Avšič-Županc, Daniel Růžek, Tizza P. Zomer, René Bødker, Thomas F. H. Berger, Sandra Martin-Latil, Nick De Regge, Alice Raffetin, Sandrine A. Lacour, Matthias Klein, Tinne Lernout, Elsa Quillery, Zdeněk Hubálek, Francisco Ruiz-Fons, Agustín Estrada-Peña, Philippe Fravalo, Pauline Kooh, Florence Etore, Céline M. Gossner, Bethan Purse

**Affiliations:** 1 Fundamental and Applied Research for Animal and Health (FARAH) Center, University of Liege, 4000 Liege, Belgium; 2 Department for Occupational Protection and Hygiene, Unit Biosafety, Biosecurity and Environmental Licences, University of Liege, 4000 Liege, Belgium; 3 UMI SOURCE, Université Paris-Saclay—UVSQ, 78000 Versailles, France; 4 ANSES, INRAE, Ecole Nationale Vétérinaire d’Alfort, UMR VIROLOGIE, Laboratoire de Santé Animale, 94700 Maisons-Alfort, France; 5 Hoogstraat 159/5, 3665 As, Belgium; 6 Centre for Infectious Disease Control, National Institute for Public Health and the Environment, 3720 MA Bilthoven, The Netherlands; 7 Ecole Nationale Vétérinaire Agroalimentaire et de l’Alimentation Nantes-Atlantique, Oniris, 44307 Nantes, France; 8 ANSES, INRAE, Ecole Nationale Vétérinaire d’Alfort, UMR BIPAR, Laboratoire de Santé Animale, 94700 Maisons-Alfort, France; 9 UMR 2000 Institut Pasteur-CNRS-Université Paris-Cité, Ecology and Emergence of Arthropod-borne Pathogens, 75015 Paris, France; 10 Animal Health Department, INRAE, 37380 Nouzilly, France; 11 UR7290: VBP: Borrelia Group, France and French Reference Centre on Lyme Borreliosis, CHRU, Unversity of Strasbourg, 67000 Strasbourg, France; 12 Institute for Infectious Diseases, University of Bern, 3001 Bern, Switzerland; 13 Oniris, INRAE, BIOEPAR, 44300 Nantes, France; 14 ANSES, Nancy Laboratory for Rabies and Wildlife, 54220 Malzéville, France; 15 Department of Epidemiology, Regional Authority of Public Health in Banská Bystrica, 497556 Banská Bystrica, Slovakia; 16 Unité Mixte de Recherché Immunorhumathologie Moléculaire (UMR IRM_S) 1109, Université de Strasbourg, INSERM, 67000 Strasbourg, France; 17 Zoonotic, Water and Foodborne Infections, The Norwegian Institute for Public Health (NIPH), 0213 Oslo, Norway; 18 Université Clermont Auvergne, INRAE, VetAgro Sup, UMR EPIA, Route de Theix, 63122 Saint-Genès-Champanelle, France; 19 Université de Lyon, VetAgro Sup, UMR CNRS 5558, Marcy-l’Etoile, 69280 Lyon, France; 20 Competence Center Climate and Health, Austrian National Institute of Public Health, 1010 Vienna, Austria; 21 Department of Virology and Immunology, National Institute for Health Development, 11619 Tallinn, Estonia; 22 Institute of Biomedicine, Department of Infectious Diseases, University of Gothenburg, 41685 Gothenburg, Sweden; 23 Institute of Microbiology and Immunology, Faculty of Medicine, University of Ljubljana, Zaloška cesta 4, 1000 Ljubljana, Slovenia; 24 Institute of Parasitology, Biology Centre of the Czech Academy of Sciences, 37005 Ceske Budejovice, Czech Republic; 25 Department of Experimental Biology, Faculty of Science, Masaryk University, 62500 Brno, Czech Republic; 26 Department of Infectious Diseases and Preventive Medicine, Veterinary Research Institute, 62100 Brno, Czech Republic; 27 Lyme Center Apeldoorn, Gelre Hospital, 7300 DS Apeldoorn, The Netherlands; 28 Animal Welfare and Disease Control, Department of Veterinary and Animal Sciences, Faculty of Health and Medical Sciences, University of Copenhagen, 1870 Frederiksberg, Denmark; 29 Agroscope, Risk Evaluation and Risk Mitigation, Schwarzenburgstrasse, 3003 Bern-Liebefeld, Switzerland; 30 Laboratory for Food Safety, ANSES, University of Paris-EST, 94700 Maisons-Alfort, France; 31 Operational Direction Infectious Diseases in Animals, Unit of Exotic and Vector-borne Diseases, Sciensano, 1180 Brussels, Belgium; 32 Reference Centre for Tick-Borne Diseases, Paris and Northern Region, Department of Infectious Diseases, General Hospital of Villeneuve-Saint-Georges, 94100 Villeneuve-Saint-Georges, France; 33 Neurologische Klinik und Poliklinik, Klinikum der Universität München, LMU München, Marchioninistraße 15, 81377 München, Germany; 34 Scientific Directorate of Epidemiology and Public Health, Sciensano, 1180 Brussels, Belgium; 35 ANSES, Risk Assessment Department, 94700 Maisons-Alfort, France; 36 Institute of Vertebrate Biology, Czech Academy of Sciences, Květná 8, 60365 Brno, Czech Republic; 37 Health & Biotechnology (SaBio) Group, Instituto de Investigación en Recursos Cinegéticos (IREC), CSIC-UCLM-JCCM, 13071 Ciudad Real, Spain; 38 Deptartment of Animal Health, Faculty of Veterinary Medicine, 50013 Zaragoza, Spain; 39 Pôle Agroalimentaire, Conservatoire National des Arts et Métiers (Cnam), 75003 Paris, France; 40 European Centre for Disease Prevention and Control (ECDC), 17183 Solna, Sweden; 41 UK Centre for Ecology & Hydrology, Benson Lane, Crowmarsh Gifford, Oxfordshire OX10 8BB, UK

**Keywords:** tick-borne encephalitis (TBE), flavivirus, TBEV, genus *Ixodes*, *Dermacentor reticulatus*, drivers, expert elicitation, ticks, multi-criteria decision analysis (MCDA), clustering analysis, sensitivity analysis, uncertainty

## Abstract

Tick-borne encephalitis (TBE) is a viral disease endemic in Eurasia. The virus is mainly transmitted to humans via ticks and occasionally via the consumption of unpasteurized milk products. The European Centre for Disease Prevention and Control reported an increase in TBE incidence over the past years in Europe as well as the emergence of the disease in new areas. To better understand this phenomenon, we investigated the drivers of TBE emergence and increase in incidence in humans through an expert knowledge elicitation. We listed 59 possible drivers grouped in eight domains and elicited forty European experts to: (i) allocate a score per driver, (ii) weight this score within each domain, and (iii) weight the different domains and attribute an uncertainty level per domain. An overall weighted score per driver was calculated, and drivers with comparable scores were grouped into three terminal nodes using a regression tree analysis. The drivers with the highest scores were: (i) changes in human behavior/activities; (ii) changes in eating habits or consumer demand; (iii) changes in the landscape; (iv) influence of humidity on the survival and transmission of the pathogen; (v) difficulty to control reservoir(s) and/or vector(s); (vi) influence of temperature on virus survival and transmission; (vii) number of wildlife compartments/groups acting as reservoirs or amplifying hosts; (viii) increase of autochthonous wild mammals; and (ix) number of tick species vectors and their distribution. Our results support researchers in prioritizing studies targeting the most relevant drivers of emergence and increasing TBE incidence.

## 1. Introduction

In Eurasia, the most common viral tick-borne disease in humans is tick-borne encephalitis (TBE), caused by a flavivirus (TBEV), which is transmitted by the bite of infected hard ticks found in woodland habitats [1]. In Europe, the most important tick vector is *Ixodes ricinus*, whereas in Russia and Asia, it is *Ixodes persulcatus*. In addition, in Asia, *Haemaphysalis concinna* also seems to play a major role [2,3]. 

Less frequently, other routes of transmission occur [4,5,6], which involve mostly the consumption of unpasteurized milk/milk products originating from infected animals or, alternatively, potential pathways such as handling infected material, blood-borne infection, and solid organ transplantation [5]. 

Vector-borne transmission of TBEV involves hard ticks [1], which transmit TBEV to a variety of small and large mammals, birds, and humans [7,8]. Each tick life stage has a preference for, or most often uses, certain vertebrate groups (often dependent on the body size of these hosts) [1]. In addition, infected migratory birds are suspected of carrying the virus to new suitable areas (e.g., [8,9]). TBEV is transmitted within ticks mainly through trans-stadial [10] and occasionally trans-ovarial transmission [11]. The importance of trans-stadial transmission is related to the fact that hard ticks take only one blood meal per life stage. In addition, co-feeding transmission (i.e., a non-viremic transmission) is now widely accepted for TBEV and occurs when infected and uninfected vectors feed in spatiotemporal proximity to each other on the same host [12]. Indeed, efficient circulation of the TBEV in tick populations is achieved by naive ticks taking a blood meal from viraemic animal hosts (systematic transmission) as well as by co-feeding [13,14]. The TBE incubation period is known to depend in the laboratory on the virus inoculation doses, virus subtypes, and host innate and adaptive immunity. In the case of vector-borne transmission, the human incubation period of TBE is usually between 7 and 14 days (range: 2 to 28 days with a median of 8 days) [15], but in the case of foodborne TBE, the median incubation period is shorter (3.5 days), and neuro-invasive disease is more common compared to vector-borne transmission [15] (reported as 38.9% in [4]). When the human infection is symptomatic, the clinical course is frequently biphasic, with influenza-like symptoms (i.e., fever, fatigue, headache, muscle aches, and nausea) and signs that range from mild meningitis (inflammation of the membrane that surrounds the brain and spinal cord) to severe meningoencephalitis (inflammation involving the brain) with or without paralysis [16,17]. After acute TBE, in up to 50% of patients, a post-encephalitic syndrome can develop, causing long-lasting morbidity that often affects the quality of life [16]. The burden of human TBE can be assessed by the calculation of the DALYs (disability-adjusted life years, i.e., the loss of the equivalent of one year of full health). From an individual perspective, DALYs of 0.23 per TBE case (95% uncertainty interval: 0.22–0.24) were estimated in Slovenia in 2009–2013, where neurological sequelae of TBE had the largest impact on the overall DALY measurement [18]. In Sweden, the social burden of TBE (cost of illness and death) has been estimated at an annual cost of €24.5 million over the 2015–2019 period [19]. 

TBEV is tentatively grouped into seven subtypes, mostly according to their phylogenetic relationships. Indeed, virus strains that differ by less than 10% of nucleotides in the polyprotein-coding gene are provisionally proposed to belong to the same subtype [20]. Each subtype can be distinguished by its different phylogeography, ecology, virulence, and pathogenicity [21]. Within the TBEV viral species, three main subtypes are already defined by the International Committee on Taxonomy of Viruses: the European TBEV (TBEV-EU), the Siberian TBEV (TBEV-Sib), and the Far Eastern TBEV (TBEV-FE), of which the TBEV-EU is predominant in Europe [22]. The co-circulation of different subtypes was already demonstrated in a hyperendemic area [23].

There is no specific antiviral treatment for TBE; the human TBE risk can be reduced by using tick repellents on skin and clothing, personal protective clothing, pasteurization of milk, and vaccination of individuals [16]. The list of the available inactivated vaccines was recently reviewed by Kubinski et al. [24]. Increasing vaccination among all age groups can be the most effective and efficient strategy to reduce the burden of TBE on endemic areas and protect the health of the whole population [25]. TBE vaccines are effective, well tolerated, and cost-effective in endemic countries [26]. Currently, there are no vaccines available for veterinary use. However, proof of concept of the immunogenicity of a new adjuvanted TBEV vaccine was demonstrated in an in vivo mouse model [27]. If this low-cost vaccine could be validated, its use in animals, especially ruminants, may mitigate food-borne transmission through raw-milk consumption.

In Europe, TBE is endemic in several central, northern, and eastern countries. Some countries experienced novel autochthonous TBE in humans, like the Netherlands in 2016 [28], Belgium in 2018 and 2020 [29,30], and the United Kingdom in 2019 [31]. Since 2017, there has been a gradual increase in reported TBE cases in the European Union/European Economic Area (EU/EEA) (see also the atlas of diseases from ECDC at the following address: https://atlas.ecdc.europa.eu/public/; accessed on 15 March 2023). In 2020, 3817 cases of TBE were reported (97.8% confirmed mainly by the detection of specific IgM and IgG antibodies in serum and cerebrospinal fluid, usually by enzyme-linked immunosorbent assay, and some after which a TBEV-specific virus neutralization test was applied) in 24 countries of the EU/EEA, with a clear seasonal pattern (95% of TBE human cases occurring between May and November and 5% between December and April) [32]. This represents an incidence of 0.9 cases per 100,000 inhabitants, i.e., 50% more than the 2016–2018 baseline.

In addition, TBE cases were generally more frequently reported among men and in the age group of 45–64 years [32]. This increased notification rate should be interpreted with caution, since multiple factors may play a role in it, but the number of reported cases is likely to vastly underestimate the number of infections (as a baseline, a correction factor for underestimation of 4.5 was estimated by [33]), since the majority of infections by TBEV are asymptomatic (i.e., manifestations with mild clinical symptoms that may remain undiagnosed). Furthermore, there is no mandatory notification of TBE in many countries. Moreover, a recent 4-year prospective cohort study performed in France from 2016 to 2019 (N = 494 cases of encephalitis) indicates that TBEV makes up 8% of encephalitis cases with an identified cause and, compared to a cohort from 2007 (N = 222), a significant increase in TBEV occurred (i.e., the number of causes of TBEV amongst all causes of encephalitis in adult patients was 3/106 and 26/257 in 2007 and 2016–2019, respectively; Fisher’s exact test, *p*-value = 0.013) [34].

An emerging infectious disease is defined by the United States National Institute of Allergy and Infectious Diseases (NIAID; https://www.niaid.nih.gov/research/emerging-infectious-diseases-pathogens (accessed on 15 March 2023)) as “infectious diseases that have newly appeared in a population or have existed but are rapidly increasing in incidence or geographic range, or that are caused by one of the NIAID category A, B, or C priority pathogens”. TBEV is within category C (emerging pathogens that could be engineered for mass dissemination in the future because of their availability, ease of production and dissemination, potential for high morbidity and mortality rates, and major health impact). Tick-borne encephalitis is also included in the WHO group of vaccine-preventable diseases [35].

Studies on the initial emergence patterns of TBEV across Eastern Europe late last century highlighted the range of interacting social and environmental drivers potentially involved in increasing the risk for human populations (e.g., [36]) through mechanisms acting to increase tick abundance, hazards (e.g., scrub regeneration on an abandoned farmland), and human exposure in forests (e.g., increased unemployment of some groups and increased wealth and leisure for others). 

The aim of this study is to investigate, for the first time, possible drivers for the current observed emergence in Europe, including the invasion of new countries and increased incidence of TBE in humans. A multi-criteria decision analysis (MCDA) method was chosen because it allows the systematic integration of information from a range of sources [37] and aims at improving repeatability and transparency [38].

## 2. Materials and Methods

The methodology followed in this expert elicitation of knowledge is the same as that previously published by the UREAR-ULiège for other emerging/zoonotic diseases [39,40,41], but adapted by a panel of experts for TBE. Briefly, we listed 59 possible drivers grouped in eight domains and elicited forty European experts to: (i) allocate a score per driver, (ii) weight this score within each domain, and (iii) weight the different domains and attribute an uncertainty level per domain. An overall weighted score per driver was calculated, and drivers with comparable scores were grouped in several terminal nodes using a regressive tree analysis.

### 2.1. Study Objective

The objective of the study was to prioritize TBE drivers in order to understand the factors that influence the observed emergence or increased incidence of TBE in humans across Europe. Using the following algorithms at December 31, 2022: (((tick-borne encephalitis virus[Title/Abstract]) OR (TBEV[Title/Abstract]) OR (tick-borne encephalitis disease[Title/Abstract])) AND (human[Title/Abstract]) AND (Europe[Title/Abstract])) search strings were conducted in PubMed (US National Library of Medicine, National Institutes of Health). The results of the search (N = 135 articles from 1994 through 2022) identified 29 review papers; other papers were related to field/epidemiological surveys (N = 45), biology studies (N = 20), diagnosis (N = 13), experimental studies (N = 1), treatments (N = 2), vaccination (N = 8), and vectors (N = 17). If we add AND (Driver[Title/Abstract]) in the algorithm, no occurrence is found.

### 2.2. Questionnaire Design 

A questionnaire is used to determine the main drivers of the observed emergence or increasing incidence of TBE in humans. A driver was defined as a factor that has the potential to directly or indirectly precipitate (“drive”) or lead to the emergence or increasing incidence of TBE in humans. A former questionnaire created to rank drivers of the emergence of animal and zoonotic diseases [39,40,41] was adjusted to capture specific possible drivers for TBE. Overall, fifty-nine drivers were identified and classified in eight different domains (Table A1). The domains (D) were: (D1) disease/pathogen characteristics (N = 12 drivers); (D2) distance to Europe and the country of the expert (spatial-temporal scales) (N = 3 drivers); (D3) ability to monitor, treat, and control the disease (N = 11 drivers); (D4) European farm characteristics (N = 5 drivers); (D5) global change (N = 4 drivers); (D6) wildlife interface (N = 5 drivers); (D7) human activity (N = 8 drivers); and (D8) economic and trade activities (N = 11 drivers). These were formatted in an Excel^®^ (Microsoft, Redmond, WA, USA, 2016) file with one spreadsheet per domain, each domain harboring its respective drivers. Each driver had a score with its own definition, which could range from 0 to 4 (i.e., 5 modalities) or 1 to 4 (i.e., 4 modalities), and an intra-driver weight point. A spreadsheet was added, in which the 8 domains were listed with an inter-domain weight.

### 2.3. Expert Elicitation to Assess Drivers of Emergence or Increasing Incidence of TBE in Humans

An expert elicitation of knowledge was conducted, which consisted of gathering the opinions of people with recognized scientific expertise (indicated by at least one publication as first or co-author) and/or experience on TBE, TBEV, ticks, and/or tick-borne diseases (Table A2). Experts are from Europe with regional, national, or international activities, have different types of employment (government institution, university, research/scientific institution, diagnostic laboratory, or hospital), and cover different sectors relevant for TBE/TBEV (public health, animal health, environment, food safety, or laboratory diagnostic). In addition, specific attention was dedicated to capturing all disciplines related to the different domains of drivers. The number of years of professional expertise followed a normal distribution (Shapiro-Wilk test; *p*-value= 0.38), with an average of 19.9 years (standard error: 10.5).

For guidance purposes, an explanation letter accompanied the questionnaire (Appendix B). Each expert was contacted personally and responded in their own capacity (not on behalf of their institution). In order to capture the degree of variability in the experts’ knowledge, the data generated by elicitation were based on the scores provided by the experts. The elicitation was conducted over the course of two months (August–September 2022).

### 2.4. Scoring, Weighting System, and Level of Uncertainty

The elicited experts were asked to provide four types of information. First, they were asked to score the drivers (as established in Table A1). For each driver, the higher the score, the higher the driver’s chance to contribute to the emergence or increasing incidence of TBE in humans. Secondly, experts were requested to weigh each driver within a specific domain (intra-domain weight). This relative weight was determined using the Las Vegas technique [42]. Briefly, experts were given a number of points to be distributed among drivers according to their importance in the specific domain (proportional pilling). If all the drivers in a given domain had been considered equivalent by experts, each of them would have received the same score. Thirdly, the relative importance of each domain was subsequently weighted by experts (inter-domain weight). Finally, the level of uncertainty was asked at the domain level and not per driver (to reduce the length of the questionnaire to be completed by each expert), using a scale from 0 (minimal uncertainty in the scoring) to 100 (maximum uncertainty in the scoring).

### 2.5. Calculation of an Overall Weighted Score for Each Driver and the Ranking Process

To obtain the overall score per driver, an aggregation method that combined the two types of weighting (i.e., intra- and inter-domain) was used. First, the driver score (coefficients attributed by experts) was standardized by dividing it by the number of possibilities. Indeed, some drivers were allotted coefficients from 0 to 4 (5 possibilities) and others from 1 to 4 (4 possibilities). Afterwards, this standardized score was multiplied by the intra-domain weight and the inter-domain weight, as given by the expert. These results led to an overall weighted score for each driver and per expert:

(1) $${\mathrm{OWS}}_{\mathrm{Dr}i}=\;{\mathrm{SDr}}_{i}\;\times \;{\mathrm{WDr}}_{i}\;\times \;{\mathrm{WDoj}}_{j}$$

In this formula, OWS_Dr*i*_ = overall weighted score for a specific driver*_i_*; SDr_i_ = standardized score for a specific driver*_i_*; WDr_i_ = intra-domain weight for a specific driver*_i_*; WDo_j_ = inter-domain weight for a specific driver*_i_* included in a specific domain*_j_*. Furthermore, all drivers were ranked based on the median overall weighted score obtained for each driver, taking into account the answers of all the experts who answered the questionnaire. The statistical difference in the median, depending on the specific driver or the group of drivers considered, was assessed through a non-parametric Kruskal-Wallis equality-of-populations rank test and median regression analysis (Stata SE 14.2; StataCorp, College Station, TX, USA).

### 2.6. Cluster Analysis

A cluster analysis was carried out using a regression tree analysis (Salford Predictive Modeler^®^, Version 8.2, Salford Systems, San Diego, CA, USA). Since the median overall weighted score (median OWS_Dr*i*_) is a continuous variable, the aim was to obtain groups of drivers with a minimal within-group variance and comparable likelihood to play a role in the emergence or increasing incidence of TBE in humans. In addition, the statistical difference between medians after grouping drivers in clusters was assessed using a non-parametric Kruskal-Wallis equality-of-populations rank test and a median regression analysis. Indeed, each driver is characterized by a median (based on all experts’ answers) and grouped. The test highlights potential significant differences between groups in terms of driver medians after clustering.

### 2.7. Sensitivity Analysis to Test the Robustness of the Expert Elicitation

In order to identify whether the ranking of drivers of the observed emergence or increasing incidence of TBE in humans was influenced by the choice of experts, three different sensitivity analyses were performed: comparison of 10 bootstraps each (random choice of 30 experts amongst 40) with the ranking of drivers obtained with all experts elicited as reference; comparison of French experts (N = 17) or other experts (N = 23) with the ranking of drivers obtained with all experts elicited as reference; and comparison of groups of experts coming from countries with a similar level of endemicity (increasing order level of endemicity from A to E). In order to have a sufficient number of experts per group, we aggregated the first two levels, group A-B, i.e., no autochthonous cases reported and sporadic cases or low endemicity, only in a few regions (N = 29), and the last three levels, group C-D-E, i.e., low to moderate, moderate to high, and low to high endemicity (N = 11) (for the details, see Table A3). The difference between the above ranking of drivers was tested using the Pearson coefficient of correlation test. If this coefficient was close to 1 and the *p*-value was less than 0.05, the correlation between the two rankings of drivers tested was considered significant.

## 3. Results

### 3.1. Response Rate and Field of Expertise Mobilized by the Experts

Forty professionals with scientific and/or field knowledge or experience regarding TBE and TBEV were contacted, and all agreed to participate. The fields and diversity of expertise are summarized in Table A2.

### 3.2. Estimating the Overall Weighted Score and Ranking of the Observed Emergence or Increasing Incidence of TBE in Humans 

**Figure 1:**
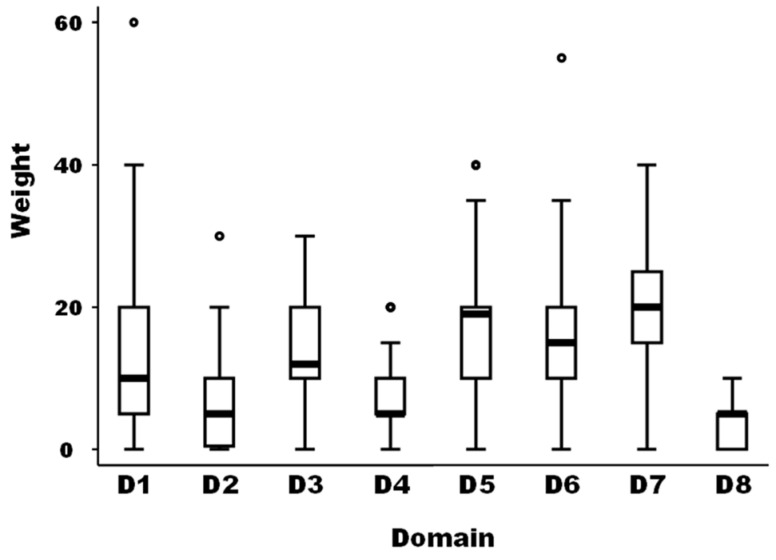
Boxplot of the relative importance of the eight domains of possible drivers of the observed emergence or increasing incidence of TBE in humans (N = 40 European experts). Legend: The bold line represents the median of the score distribution between the different experts attributed to each domain; the solid lines at the top and bottom of each rectangle represent, respectively, the first and third quartiles; adjacent lines to the whiskers represent the limits of the 95% confidence interval; small circles represent outside values. The eight domains of drivers are: D1, disease/pathogen characteristics; D2, distance to Europe and the country of the expert (spatial-temporal scales); D3, ability to monitor, treat, and control the disease; D4, European farm characteristics; D5, global change; D6, wildlife interface; D7, human activity; and D8, economic and trade activities.

The medians of the weight between the eight domains of drivers as well as for the different 59 drivers were not equal according to the non-parametric Kruskal-Wallis test (Chi-squared test = 105.5 with 7 d.f. and α = 0.05, *p*-value = 0.0001; and Chi-squared test = 785.4 with 58 d.f. and α = 0.05, *p*-value = 0.0001, for the weights between domains and the weights of the different drivers, respectively) (Figure 1). 

The median of the weights of domains D2, D4, and D8 was significantly lower than the median of domain D1 as a reference (median regression; *p*-value = 0.019). The median of the weights of domains D5 and D7 was significantly higher than the median of domain D1 as a reference (median regression; *p*-value < 0.001).

**Figure 2:**
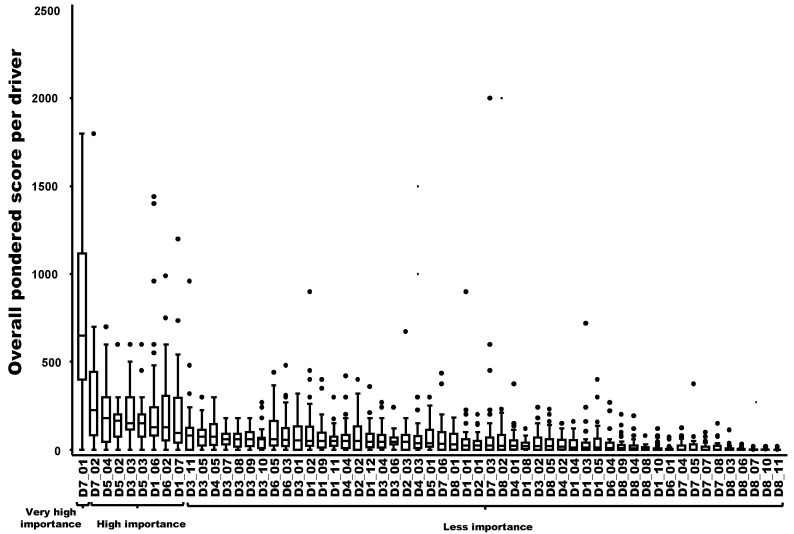
Ranking of the overall weighted score for each potential driver of the observed emergence or increasing incidence of TBE in humans (boxplot based on input from 40 European experts). Legend: The x-axis represents the drivers with the following codification: D1 to D8 refer to the eight domains of drivers, and D1_1 to D8_11 refer to a specific driver (for the codification, see Table A1). A relation to Figure 3 was provided by the group, which named “very high importance”, “high importance” and “less importance”, as possible drivers of the observed emergence or increasing incidence of TBE in humans.

Nine drivers out of 59 were ranked in decreasing order as having a very high importance (N = 1) or a high importance (N = 8) in the probability of playing a key role in the emergence or increasing incidence of the TBE in humans. The most important driver was related to changes in human behavior/activities leading to more contacts with the pathogen in high-risk areas (e.g., recreational activities, forest activities, and mushroom picking) (D7-1). The following eight drivers had a high importance in the probability of playing a key role: changes in eating habits or consumer demand leading to an increased exposure to the hazard through food (D7-2); changes of landscape, e.g., landscape fragmentation, creation of barriers, landfill sites (D5-4); the influence of humidity in the survival and transmission of the pathogen/disease (D5-2); the difficulty to control reservoir(s) and/or vector(s) (D3-3); the influence of temperature in the survival and transmission of the pathogen (D5-3); the number of wildlife compartments/groups (e.g., small mammals, birds, and ungulates) playing a role as reservoirs or amplifying hosts for the pathogen and potential spread (D1-6); the increase of autochthonous (indigenous) wild mammals in Europe and neighboring countries (D6-2); and the number of tick species vectors of the pathogen and their distribution within the country (D1-7) (Figure 2).

### 3.3. Cluster Analysis

**Figure 3:**
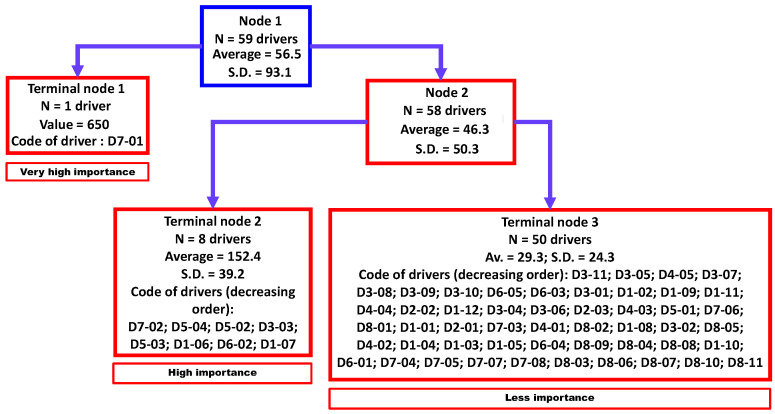
Aggregation of drivers of the observed emergence or increasing incidence of TBE in humans into three homogenous groups using a regression tree analysis. Legend: N, number; SD, standard deviation. D1-01 to D8-11 refer to a specific driver (for the codification, see Table A1).

Three clusters were identified by regression tree analysis (Figure 3) that were significantly different (non-parametric Kruskal-Wallis equality-of-populations rank test; chi-squared test = 22.6 with two degrees of freedom (d.f.) and α = 0.05; *p*-value = 0.0001). These three clusters were classified as “very high importance” (N = 1 driver), “high importance” (N = 8 drivers), and “less importance” (N = 50 drivers).

### 3.4. Sensitivity Analysis of the Impact of Experts on the Final Ranking of the Observed Emergence or Increasing Incidence of the TBE in Humans 

The result of three different sensitivity analyses indicated that, irrespective of the experts excluded, excluding some experts only had very limited or no significant effects on the ranking compared to the reference (all experts elicited). Firstly, using 10 bootstraps of 30 experts amongst 40, the Pearson coefficient of correlation between each bootstrap against the ranking of 40 experts as a reference was very high (value between 0.97 and 0.99, with a *p*-value < 0.0001). Secondly, comparing the ranking of drivers either between French experts (N = 17) or other experts (N = 23) and all experts as a reference, the Pearson coefficient of correlation was of 0.92, with a *p*-value < 0.0001. Thirdly, comparing the ranking of drivers either between experts coming from countries with no autochthonous cases reported and sporadic cases or low endemicity (N = 29) or experts coming from countries with low to moderate, moderate to high, and low to high endemicity (N = 11), and all experts as a reference, the Pearson coefficient of correlation was respectively of 0.96 and 0.90, with a *p*-value < 0.0001.

### 3.5. Level of Uncertainty Per Domain of Drivers

**Figure 4:**
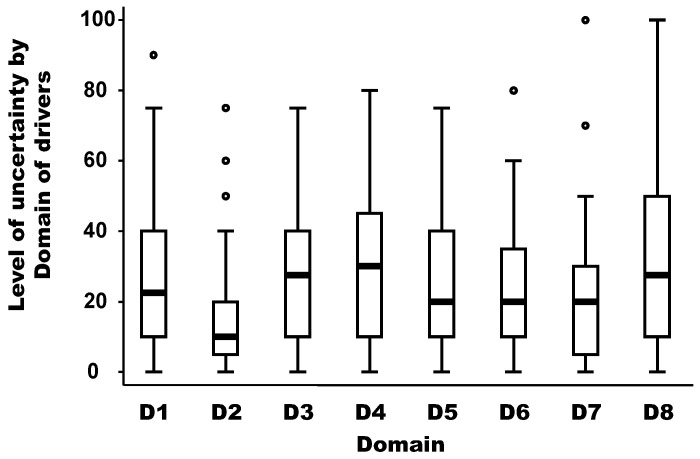
Level of uncertainty per domain of drivers. Legend: The bold line represents the median of the level of uncertainty attributed by experts using a scale from 0 (minimal uncertainty in the scoring) to 100 (maximum uncertainty in the scoring); the solid lines at the top and bottom of each rectangle represent, respectively, the first and third quartiles; adjacent lines to the whiskers represent the limits of the 95% confidence interval; small circles represent outside values. The eight domains of drivers are: D1, disease/pathogen characteristics; D2, distance to Europe and the country of the expert (spatial-temporal scales); D3, ability to monitor, treat, and control the disease; D4, European farm characteristics; D5, global change; D6, wildlife interface; D7, human activity; and D8, economic and trade activities.

The level of uncertainty per domain of drivers is depicted in Figure 4. The medians of the uncertainty between domains of drivers were not equal according to the non-parametric Kruskal-Wallis test (chi-squared test = 19.91 with 7 d.f. and α = 0.05, *p*-value = 0.018). The median of the uncertainty in the domain D2 (distance to Europe and the country of the expert) was significantly lower (around 10) than the median of other domains, which was between 20 and 30 (median regression; *p*-value < 0.02). In addition, there is no linear (Pearson coefficient correlation = −0.17; *p*-value = 0.69) or non-parametric (Spearman rank correlation = −0.39; *p*-value = 0.34) relationship between the median weight and the median uncertainty by domain.

## 4. Discussion

Fifty-nine possible drivers of the emergence or increasing incidence of TBE in humans were ranked and aggregated into three homogenous groups according to the present expert elicitation of knowledge. Only the first nine most important ranked drivers will be further discussed, with a focus on the two categorized in the regression tree as “very high importance” nodes (N = 1) and “high importance” nodes (N = 8), respectively. Moreover, the sensitivity analysis showed limited and no significant effects of the experts involved, indicating an acceptable robustness of the elicitation for drivers included in the two first terminal nodes of the tree. In addition, the median level of uncertainty for all domains of drivers was moderate (between 20 and 30 on a scale from 0 to 100) except for the domain D2, which was lower (around 10). 

The most important driver for the observed emergence or increasing incidence of TBE in humans recognized during this expert elicitation are changes in human behavior/activities leading to more contacts with the pathogen in high-risk areas (e.g., recreational activities, forest activities, and mushroom picking) (D7-1). This driver was first implicated in TBEV emergence by [36], who suggested that increased unemployment and marginal employment in forests due to the socio-economic transition were driving an increase in human TBEV exposure across eastern Europe. Other studies have linked changing human behavior to new autochthonous cases of TBE in infected areas [30,36,43]. Preventive actions are mostly related to tick bite avoidance (wearing protective clothing such as long sleeve tops and long trousers tucked into socks or boots and using tick repellents on the skin and/or clothing) [35]. More studies are needed to understand how the risk of tick bite exposure varies between different types of recreational and forest activities [44], to refine and tailor information and awareness campaigns for the general population or for particularly high-risk social groups (e.g., [45]).

The second important driver is the change in eating habits or consumer demands, leading to an increased exposure to hazards through food (D7-2). Although it is not well documented, there is a trend toward consuming minimally processed and locally produced foods, such as raw milk and certain raw milk cheeses. Slovaks consume traditional products made from raw sheep milk; however, sheep milk is often mixed with goat milk for higher yield, which increases the risk of TBEV milk infection. In Slovakia, the alimentary route of TBEV transmission is responsible for up to 20% of all reported human TBE cases [6]. Preferences for raw milk/milk products like cheese have changed over the last decade and vary between countries. In the past, consumers considered raw milk cheese to be less safe [46]. However, cultural determinants should also be considered, like pro-raw milk consumers in France who focus on the traditional and authentic character of the product [47] and in Switzerland who focus on positive health effects like allergy protection and support of the gut microbiome [48] or the international FACEnetwork (Farmhouse and Artisan Cheese and Dairy Producers European Network, www.face-network.eu (accessed on 15 March 2023)). There are also scientific data suggesting that raw milk consumption is associated with the prevention of atopy (i.e., an exaggerated IgE-mediated immune response) (e.g., see the PASTURE European Project [49]). Moreover, as explained by some stakeholders in a recent report [50], the consumption of raw milk products varies across cultures. Currently, raw milk is cheap compared to pasteurized milk and considered a standard product; the perception of healthy products is another aspect, and people increasingly look for these kinds of food products [50]. More studies are needed to estimate the consumption and safety (specifically for TBE) of raw milk and raw milk products by European consumers, as well as their contamination by the TBEV.

A third driver identified in this study is a landscape change, e.g., landscape fragmentation, the creation of barriers, and landfill sites (D5-4). The importance of landscape predictors of TBE was already identified within diverse frameworks like the link between land cover, land use, and land ownership and reported TBE incidence in rural parishes [43] or the hazard related to functional resources in the landscape determining where the reservoirs, vectors, and viruses are present, and exposure as the attractiveness and accessibility of people to hazardous areas and habitats [51]. Given the diverse and rapid land use change processes affecting the regions in which TBEV is circulating, it is critical to understand the joint socio-ecological mechanisms and the drivers of interactions between wildlife and domestic vertebrate reservoirs, ticks, and people that link hazards, exposure, and risk to landscape structure. 

The fourth and sixth drivers, in order of importance, are the influence of humidity (D5-2) and temperature (D5-3), respectively, on the survival of the vector and consequently successful (or increased) transmission of TBEV. The main vectors of TBEV are hard ticks of the genus *Ixodes* (especially *Ixodes ricinus* across Europe and *Ixodes persulcatus* in north-eastern regions). The habitat suitability for the vector (suitable sites for breeding, questing, and resting) is influenced by the relative humidity and temperature, and these parameters contribute as predictor data for modeling the risk for tick-borne diseases like TBE [52,53]. Several laboratory experiments demonstrated the importance of humidity and temperature for tick survival (e.g., [54,55,56]). Both *I. ricinus* and *I. persulcatus* are vulnerable to desiccation. Therefore, these ticks require a relative humidity above 80% in their microhabitats to quest and survive [57,58]. The microclimatic temperature is another key driver of vector-borne disease transmission, as replication of arboviruses within the poikilothermic vectors is highly dependent on environmental temperature [59,60]. Indeed, the extrinsic incubation period and the blood meal digestion period are influenced by the temperature surrounding the vectors [60,61,62]. Additional microclimatic data are needed for a more accurate prediction of vector-borne diseases like TBE [61]. The impact of climate change on the risk of TBEV transmission is a question of interest. For example, a rise in temperature can increase the length of the tick questing season and, depending on the context, can also increase the numbers of susceptible ticks (larvae and nymphs) and the number of infected nymphs co-feeding on the same hosts, resulting in increased human infections [62]. Furthermore, a rise in temperature can increase the spread of ticks to areas with higher elevations (e.g., [63,64]) and may also drive changes in human behavior [65]. Theories explaining the consequences of climate change on tick-borne diseases in Europe, including TBE, are discussed in a recent review paper (and include effects on the intra-stage development of ticks, an extended transmission season, changes in the ecological balance of species abundance and interactions in the ecosystem, and climate-related range expansion of vectors, reservoir hosts, or human populations) [66].

However, more studies are needed to accurately predict the distribution of ticks and TBE. Tick-borne disease risk models need to be developed at a fine enough spatial resolution to capture the focal distribution of TBEV within the range of its widespread *Ixodes* vector and need to integrate climate and landscape drivers and the dynamics of wildlife and domestic hosts and people, aligning with a One Health perspective, and capitalize on advances in digital epidemiology and artificial intelligence (e.g., [67,68,69]) and citizen science approaches using, e.g., apps to collect data [70].

The fifth and seventh drivers were, respectively, the difficulty of controlling reservoir(s) and/or vector(s) (D3-3) and the number of wildlife compartments/groups (e.g., small mammals, birds, ungulates) playing a role as reservoirs or amplifying hosts for the pathogen and its potential spread (D1-6). Host vertebrates susceptible to TBEV (i.e., that can replicate TBEV with or without expression of clinical disease) with high viraemia (rodents (*Apodemus*, *Myodes*, *Micrototus*, *Micromys*, *Pitimys*, and *Arvicola*), insectivores like hedgehogs (*Erinaceus europaeus*) and moles (*Talpa europea*)) [71] or prolonged viraemia (some rodents like the red vole (*Myodes rutilus*) [72,73], and the bank vole (*Myodes glareolus*) [74,75]) play a key role in the transmission of TBEV to ticks [17] and co-feeding hosts [10]. Foxes (*Vulpes vulpes L*) can also be experimentally infected by infestation with TBE-infected ticks; these foxes became viraemic and suffered from encephalitis and paralysis [76]. Ticks (*Ixodes ricinus*, *Ixodes canisuga*, and *Ixodes hexagonus*) are able to infest red foxes [77], and TBEV was isolated from two adult *I. ricinus* and *I. hexagonus* ticks [78]. In addition, a first occurrence of clinical encephalitic manifestations due to TBEV in a roe deer (*Capreolus capreolus* L.) [79] and in a horse [80] was recently documented in Europe, suggesting that TBEV should be included in the differential diagnosis of roe deer and horses presenting neurologic disorders. It is critical to understand the relative role of these diverse hosts in transmission in different parts of the range of TBEV in order to predict further spread and impacts and design appropriate mitigation. Moreover, the control of reservoir hosts seems to be very difficult (e.g., [81]), and the control of ticks is currently impossible. In addition, seasonal bird migration has been identified as a risk factor for TBEV spread since birds are known to transport ticks and tick-borne pathogens such as TBEV [8,82].

The eighth most important driver identified is the increase of autochthonous (indigenous species) wild mammals in Europe and neighboring countries (D6-2). The wild mammal inventory was recently updated for some species and revealed an increase in abundance/range, driven by conservation efforts [83,84]. Such a trend should have some consequences for ticks and tick-borne diseases. As an example, the spatio-temporal increases in Western roe deer (*Capreolus capreolus*) in Europe should increase deer density and the density of bloodmeal sources for seeking ticks, which could potentially amplify tick populations [85,86]. A higher abundance of large mammals may lead to a greater tick population but also to other effects, such as dilution of the acarologic risk, predation on competent or non-competent hosts, or competition with specialized predators [87,88,89]. According to the country and scale of study, the abundance of ungulates or carnivores correlated either positively or negatively with the number of TBE cases (e.g., [87,89,90]). Moreover, few hard data on distribution, abundance, tick infestation rates, and TBEV infection rates are available for small rodent population dynamics, a species that is susceptible to TBEV with a high viremia [17]. More studies are needed to fill this gap or to investigate surrogate data (e.g., food supply during winter increases survival of small rodents [91] or breeding success of barn owls (*Tyto alba*) reflects the trends of small rodent populations [92]) to allow trend observation or trend analysis. 

The ninth most important driver is the number of tick species that are competent vectors of the pathogen and their distribution in the country (D1-7). Although more than 14 species of ticks can be infected by TBEV [93], *I. ricinus* and *I. persulcatus* ticks are considered to be the main vectors of the European subtype and the Siberian and Far Eastern subtypes of TBEV [17,88,93]. However, the additional potential role of *Dermacentor reticulatus* was recently evidenced in experimental [94] and field [95] conditions. *Dermacentor reticulatus* is frequently found on pets, mainly on dogs (their movements without tick control can contribute to the spread of ticks) and is active in the winter or during the colder months [96,97,98]. Its range has also expanded in Europe [99,100,101,102]. Such a discovery deserves attention in order to better understand the eco-epidemiology of TBEV in relation to the trend of increasing human TBE cases, particularly since these ticks regularly feed on domestic ruminants, which may then transmit TBEV through their milk. The known European distribution of *I. ricinus*, *I. persulcatus,* and *D. reticulatus* ticks is available on a dedicated online platform [103,104].

Possible limitations exist with experts’ elicitation of knowledge (for a review, see [105]), like cognitive bias, overconfidence, or responses being conditioned by the recent high-profile findings (e.g., TBEV in unpasteurized milk) rather than taking a longer-term view. In this elicitation, the sensitivity analysis addresses these limitations to some degree. In addition, expert elicitation of knowledge is complementary and not in opposition to or a substitute for empirical One Health studies that address social and environmental drivers of risk in different geographical areas of emergence.

## 5. Conclusions

Scientific knowledge on possible drivers of the observed emergence or increasing incidence of TBE in humans is scant. In this context, expert elicitation of knowledge and multi-criteria decision analyses, in addition to clustering and sensitivity analyses, allowed the identification of nine drivers of either very high or high importance. These drivers should be the focus of future studies so we can increase and refine our understanding of the epidemiology of TBE in Europe and support decision-making to reduce TBEV exposure and its impacts.

## Data Availability

The data that support the findings of this study are available from the corresponding authors upon reasonable request.

## Appendix A

**Table A1:** Domains of Each Defined Driver and Their Respective Defined Coefficients (Scores).

DOMAIN D1. DISEASE/PATHOGEN CHARACTERISTICS.		
D1-1	Current knowledge of the pathogen (e.g., transmission routes, incubation period depending of the transmission route, infectious dose, etc.).	
	Score 0	
	Score 1	Very high: deep scientific knowledge on the pathogen, extensive scientific literature available on its biology (transmission mode, infectivity, etc.).
	Score 2	High: detailed scientific knowledge on the pathogen but conflicting scientific results; some elements of the pathogen’s biology are still not elucidated.
	Score 3	Moderate: limited scientific knowledge on the pathogen agent because it is still under characterization; pathogen recently discovered/isolated but belonging to a well-known and studied family of pathogens; the pathogen is characterized by multiple variants that are not characterized yet.
	Score 4	Low: lack of scientific knowledge on the pathogen (multiplication, infectivity, incubation period, transmission mode, transmission route, etc.); pathogen agent recently discovered and emerging.
D1-2	The current domestic species able to excrete the virus into the milk.	
	Score 0	
	Score 1	Low: only one species is involved.
	Score 2	Medium: two species involved.
	Score 3	High: three species involved.
	Score 4	Very high: more than three species involved.
D1-3	Genetic variability with time of the infectious agent.	
	Score 0	Negligible: the infectious agent is genetically stable.
	Score 1	Low: the genetic variability is low; therefore, it has a low effect in the (re-)emergence of the pathogen.
	Score 2	Medium: the pathogen is considered to have a medium genetic variability.
	Score 3	High: the pathogen is considered to have a high genetic variability.
	Score 4	Very high: very high genetic variability (e.g., high mutation rate and re-assortment and recombination).
D1-4	Risk of showing no clinical signs and silent spread during infection and post-infection in humans.	
	Score 0	Null: silent spread is not a part of pathogen’s characteristics.
	Score 1	Low: very short incubation period and signs of infections that are easily detected/recognised.
	Score 2	Moderate: very short incubation period and signs of infection that are NOT easily detected/recognised.
	Score 3	Medium: long incubation period, clinical signs that are not characteristic, and therefore specific diagnosis is necessary to detect infection.
	Score 4	Very high: long incubation period. Disease/infection shows not clinical symptoms during the infectious period; chronic shedder.
D1-5	Existence of susceptible/ vulnerable groups (people at a higher risk of developing symptomatic or severe forms of the disease) in relation to its influence on the prevalence/incidence of the disease in your country.	
	Score 0	
	Score 1	Negligible: no known susceptible groups. The disease affects all populations and the increasing prevalence/incidence of vulnerable groups (elderly nd immuno-suppressed people), has no effect on the (re-)emergence or increasing incidence of the disease in your country.
	Score 2	Low: an increase in the prevalence/incidence of vulnerable groups (elderly and immuno-suppressed people) has a low effect on the (re-)emergence or increasing incidence of the disease in your country.
	Score 3	Moderate some populations are at a higher risk of developing symptomatic forms of the disease and the increasing prevalence/incidence of vulnerable groups (elderly and immune suppressed people) have a moderate effect on the (re-)emergence or the increasing incidence of the disease in your country.
	Score 4	High: some populations are at a higher risk of developing severe forms of the disease and the increasing prevalence/incidence of vulnerable groups (elderly and immune suppressed people) have a high effect on the (re-)emergence of the disease in your country.
D1-6	The number of wildlife compartments/groups (e.g., small mammals, birds, ungulates, etc.) playing roles as reservoirs or amplifying hosts for the pathogen and its potential spread.	
	Score 0	Null: no known wildlife reservoir. The pathogen has never been reported in wildlife species (i.e., possible detection but not necessary proof of reservoir or amplification host).
	Score 1	Low: the pathogen has been reported in only one group of wildlife.
	Score 2	Moderate: the pathogen has been reported in two groups of wildlife.
	Score 3	High: the pathogen has been reported in at least three groups of wildlife.
	Score 4	Very high: disease establishes itself in wildlife as a reservoir and is very hard to eradicate from wildlife. Livestock easily infected via wildlife contact; or the pathogen has never been investigated in wildlife species.
D1-7	Number of tick species vectors of the pathogen and their distribution in the country.	
	Score 0	Null: no known tick.
	Score 1	Low: only one species of tick is present in the country but its role in the transmission is presumed low (has not been assessed to date) or has a limited distribution in the country.
	Score 2	Medium: more than one species of tick are present in the country but their role in the transmission are presumed low (has not been assessed to date) or have a limited distribution in the country.
	Score 3	High: only one competent tick is present and can carry and spread the disease and is found spread in most of the territory.
	Score 4	Very high: more than one species of tick can carry and spread the disease and are found spread in most of the territory.
D1-8	Existence of other vectors, including mechanic vectors (e.g., mosquitoes, midges, and culicoides) and a potential spread.	
	Score 0	Null: no other known vector.
	Score 1	Low: only one type of vector is present in the country but its role in the transmission is presumed low (has not been assessed to date).
	Score 2	Moderate: more than one type of vector exists in the country but their role in the transmission is presumed low and has only been suspected as a source and spread of the disease.
	Score 3	High: only one vector is present in the country and its role in transmission is presumed moderate to high.
	Score 4	Very high: more than one type of vector can carry and spread the disease and are found spread in most of the territory.
D1-9	Transmission of the pathogen to humans.	
	Score 0	
	Score 1	Low: humans are infected by direct close contact with other infected humans and by vertical transmission.
	Score 2	Moderate: transmission by direct and indirect contacts only (e.g., through vehicles, foodborne, clothes, and instruments) or non-flying vector (e.g., ticks).
	Score 3	High: vector transmission by flying vectors (e.g., culicoides and mosquitoes).
	Score 4	Very high: more than three modes of transmission and/or airborne transmission.
D1-10	Environmental persistence.	
	Score 0	Null: pathogen does not survive in the environment.
	Score 1	Low: only anecdotal isolation of the pathogen from the environment has been recorded.
	Score 2	Moderate: the survival of the agent in the environment is limited (only temporary) and it is dependent on certain environmental conditions (e.g., humidity, temperature, rainfall, etc.).
	Score 3	High: the survival of the agent in the environment is limited (only temporary) and NOT dependent on certain environmental conditions (e.g., humidity, temperature, rainfall, etc.).
	Score 4	Very high: agent naturally surviving in the environment (soil and water) and organic materials is were it has a long-term survival.
D1-11	Transmission of the pathogen through food.	
	Score 0	
	Score 1	Low: transmission through only one food category (e.g., raw milk).
	Score 2	Moderate: transmission through two food categories (e.g., milk and cheese).
	Score 3	High: transmission through three food categories (e.g., milk, cheese, and meats).
	Score 4	Very high: transmission through more than three food categories.
D1-12	Virus persistence in food and/ or during food processing.	
	Score 0	Null: pathogen does not survive in dairy products.
	Score 1	Low: only one anecdotal isolation of the pathogen from dairy products has been recorded.
	Score 2	Moderate: the survival of the agent in the environment (matrix) is limited (only temporary) and it is dependent on certain environmental conditions, such as humidity, temperature, rainfall, etc.
	Score 3	High: the survival of the agent in dairy products is limited and dependent on the physico-chemical characteristics of the product (e.g., pH and aw) and the food processing technology (e.g., pasteurization, sterilization, and ripening).
	Score 4	Very high: agent naturally resistant to food processing technologies (long term-survival).
Number of drivers = 12, hence 120 points to be distributed across this domain for the intra-domain weighing.		
DOMAIN D2. DISTANCE TO EUROPE AND YOUR COUNTRY.		
D2-1	Current incidence (cases)/prevalence of the disease in the world, excluding Europe (the case of Europe is considered in the next criteria).	
	Score 0	
	Score 1	Pathogen has been reported only in the countries of the Australasia (Australia, New Zealand, New Guinea, and neighbouring Pacific Islands) region.
	Score 2	Disease was reported in countries of the Americas, the Caribbean, and Asia (excluding the Russian Federation).
	Score 3	Disease was reported/present in the African continent.
	Score 4	Disease was reported in countries of the Mediterranean Basin, Middle East, and the Russian Federation.
D2-2	European geographic proximity of the pathogen/disease to your country.	
	Score 0	
	Score 1	Disease has never been present in Europe.
	Score 2	Disease has been reported in Europe in the past but is currently exotic.
	Score 3	Disease is currently present in at least one European country which is NOT bordering your country.
	Score 4	Disease is currently present in at least one of the countries bordering your country.
	Score 5	Disease reported in the country (human cases and/or infections in animals).
D2-3	To your knowledge when was the disease last reported in Europe.	
	Score 0	More than 20 years ago.
	Score 1	More than 10 years ago.
	Score 2	More than 5 years ago.
	Score 3	More than 1 year ago.
	Score 4	Currently present in Europe.
Number of drivers = 3, hence 30 points to be distributed across this domain for the intra-domain weighing.		
DOMAIN D3. ABILITY TO MONITOR, TREAT, AND CONTROL THE DISEASE.		
D3-1	Ability of preventive/control measures to stop the disease from entering the country or spreading (containment of the epidemic/pandemic), EXCLUDING treatment, vaccination, and vector(s)/reservoir(s) control.	
	Score 0	
	Score 1	Very High: sanitary certificate; effective traceability of animals and by-products; effective disinfection measures; no contact between domestic and wild animals; and effective biosecurity measures.
	Score 2	High: no sanitary certificate; effective traceability of animals and by-products; effective disinfection measures; limited or incomplete possibilities to restrict contacts between domestic and wild animals; and effective biosecurity measures.
	Score 3	Low: no sanitary certificate; incomplete traceability of animals and by-products; ineffective disinfection measures; incomplete restriction of contacts between domestic and wild animals; incomplete restriction of wildlife movements; and ineffective biosecurity measures.
	Score 4	Very low: no sanitary certificate; no traceability of animals and by-products; ineffective disinfection measures; impossibility to restrict spread by wild animals; and totally ineffective biosecurity measures.
D3-2	Vaccine availability for humans.	
	Score 0	
	Score 1	Very high: commercialized vaccine available on a global scale (worldwide).
	Score 2	High: local/mono-strain vaccine available at a regional/national scale (not systematically available for a global fight plan).
	Score 3	Low: experimental vaccine, not commercialized to date; severe adverse reaction when applied; limited protector effect.
	Score 4	Very low: absence; no vaccine available on the market for a use in the species considered in the study, no experimental vaccine either.
D3-3	Control of reservoir(s) and/or vector(s).	
	Score 0	Null: no vector-borne transmission and/or no reservoir(s) known to date.
	Score 1	Very high: effective; limited reservoir(s) with limited geographical repartition, easy-to-identify; high scientific knowledge on vector(s)/reservoir(s); effective fighting measures.
	Score 2	High: limited reservoir(s)/vector(s) with limited geographical repartition; easy-to-identify, high scientific knowledge on vector(s)/reservoir(s); effective fighting measures but NOT applicable at a large scale; limited fighting measures.
	Score 3	Low: numerous reservoirs vectors identified with limited geographical repartition; hard to identify; lack of scientific knowledge on vector(s)/reservoir(s); fighting measures are not adequately effective—resistances and/or negative impact on environment.
	Score 4	Very low: numerous vector(s)/reservoir(s)identified with wide geographic distribution; hard to identify, absence of scientific knowledge on vector(s)/reservoir(s); NO effective fighting measure against vector(s) (no active molecule, resistance to measures applied).
D3-4	Detection of pathogen circulation in humans—e.g., difficulties for the physicians to diagnose the disease or clinical signs that not so evident.	
	Score 0	
	Score 1	Very high: disease is easily detected with pathognomonic clinical signs and physicians are aware of the disease and willing to notify it as soon as possible it.
	Score 2	High: disease is easily detected by the clinical signs but physicians do not have sufficient knowledge/awareness nor interest to notify it or there is no central system of notification.
	Score 3	Moderate: disease is not as easily detect by the clinical signs and physicians do not have sufficient knowledge/awareness nor interest to notify or there is no central system of notification.
	Score 4	Low: humans do not show any pathognomonic clinical sign(s); physicians are reluctant to declare/notify or there is no central system of notification.
D3-5	Detection of pathogen circulation in domestic animals—e.g., difficulties for the farmer/veterinarian to report the disease or clinical signs that are not so evident.	
	Score 0	
	Score 1	Very high: disease is easily detected with clinically signs and farmers are aware of the disease and willing to report it as soon as possible.
	Score 2	High: disease is easily detected by the clinical signs but farmers do not have sufficient knowledge/awareness nor interest to report it.
	Score 3	Moderate: disease is not as easily detect by the clinical signs and farmers do not have sufficient knowledge/awareness nor interest to report it.
	Score 4	Low: the infected animal does not show any pathognomonic clinical sign(s); farmer is reluctant to declare/report any abnormality.
		
D3-6	Methods for detecting viral agents in human in your country.	
	Score 0	
	Score 1	Very High: field tests are available and easy to use, with highly discriminating sensitivity and specificity (including infectivity tests).
	Score 2	High: tests are used in local/regional laboratories but not in the field.
	Score 3	Low: tests are only used in *specialized* laboratories/national reference laboratories.
	Score 4	Very Low: there are no detection methods available to date.
D3-7	Methods for detecting viral agent in animals in your country.	
	Score 0	
	Score 1	Very High: field tests are available and easy to use, with highly discriminating sensitivity and specificity (including infectivity tests).
	Score 2	High: tests are used in local/regional laboratories but not in the field.
	Score 3	Low: tests are only used in *specialized* laboratories/national reference laboratories.
	Score 4	Very Low: there are no detection methods available to date.
D3-8	Methods for detecting viral agent in ticks in your country.	
	Score 0	
	Score 1	Very High: field tests are available and easy to use, with highly discriminating sensitivity and specificity (including infectivity tests).
	Score 2	High: tests are used in local/regional laboratories but not in the field.
	Score 3	Low: tests are only used in *specialized* laboratories/national reference laboratories.
	Score 4	Very Low: there are no detection methods available to date.
D3-9	Methods for detecting viral agent in food in your country.	
	Score 0	
	Score 1	Very High: field tests are available and easy to use, with highly discriminating sensitivity and specificity (including infectivity tests).
	Score 2	High: tests used in local/regional laboratories but not in the field.
	Score 3	Low: tests are only used in *specialized* laboratories/national reference laboratories.
	Score 4	Very Low: there are no detection methods available to date.
D3-10	Disease in humans that is currently under surveillance overseas (OMS and EU).	
	Score 0	
	Score 1	Very high: generalized surveillance implemented by ALL EU Member States and the worldwide surveillance.
	Score 2	High: surveillance of the pathogen in EU member states only.
	Score 3	Low: surveillance only in some EU member states (because they had cases of the disease) and only in some NON-EU countries (not a disease reported in any international organisations).
	Score 4	Very low: absence of surveillance of the pathogen in ALL EU member countries AND worldwide.
D3-11	Eradication experience in other countries and/or your country.	
	Score 0	
	Score 1	Very high: previous experience on eradication has been applied rapidly and successfully.
	Score 2	High: previous experience on eradicating the disease but with some setbacks in the process.
	Score 3	Low: knowledge on eradication procedures but have never had to implement an eradication program in your country.
	Score 4	Very low: it is an endemic disease with the impossibility of eradication or is a novel disease; first time countries are faced with a new disease to eradicate.
Number of drivers = 11, hence 110 points to be distributed across this domain for the intra-domain weighing.		
DOMAIN D4. Farm/European characteristics.		
D4-1	Type of farms / productions: dairy / beef (cattle) production, sheep or goat production, mono-species farms—one single farmed animal (e.g., only bovines) or multi-species farms (farms with more than one species, e.g., goats and bovines in the same farm/land/premises).	
	Score 0	
	Score 1	Negligible: the type of farm does not influence human disease incidence in your country.
	Score 2	Low: the type of farm has a low effect on human disease incidence in your country.
	Score 3	Moderate: the type or types of farmed animals has a moderate effect on human disease incidence in your country.
	Score 4	High: the type of farmed animals has a high influence on human disease incidence in your country.
D4-2	Animal density of farms. Extensive (small holders with a few animals) *vs.* intensive farming—effect on the human contamination through animal products (e.g., consumption of unpasteurised milk products).	
	Score 0	
	Score 1	Negligible: animal farm density has a negligible effect on human disease incidence in your country.
	Score 2	Low: farm density (extensive or intensive) of animals has a low effect on human disease incidence in your country.
	Score 3	Moderate: farm density of animals in the farm (extensive v/s intensive) has a moderate effect on human disease incidence in your country.
	Score 4	High: farm density of animals has a high effect on human disease incidence in your country.
D4-3	Farm management practices (off-ground farming, treatment, feeding practices, etc.)	
	Score 0	
	Score 1	Negligible: management practices have a negligible effect on human disease incidence in your country.
	Score 2	Low: management practices have a low effect on human disease incidence in your country.
	Score 3	Moderate: management practices have a moderate effect on human disease incidence in your country.
	Score 4	High: management practices have a high effect on human disease incidence in your country.
D4-4	The number of animals infected by TBEV in the herd (infection rate).	
	Score 0	
	Score 1	Negligible: the number of animals infected by TBEV in farms have a negligible effect on human disease incidence in your country.
	Score 2	Low: the number of animals infected by TBEV in farms have a low effect on human disease incidence in your country.
	Score 3	Moderate: the number of animals infected by TBEV in farms have a moderate effect on human disease incidence in your country.
	Score 4	High: the number of animals infected by TBEV in farms have a high effect on human disease incidence in your country.
D4-5	The rural(farm)-wildlife interface.	
	Score 0	
	Score 1	Negligible: the disease has never (re-)emerged from the narrowing of the farm-wildlife interface.
	Score 2	Low: the disease has a low probability to (re-)emerge via the livestock farm-forest interface. The disease has been known to (re-)emerge from the wild bush, but very rarely.
	Score 3	Moderate: the disease has a moderate probability to occur via the farm/wildlife interface. Barriers (natural or artificial) are needed to keep the disease incidence in livestock.
	Score 4	High: there is a high probability for the disease to (re-)emerge via the farm/forest interface. Barriers (natural or artificial) separating farms from natural forests are ineffective.
Number of drivers = 5, hence 50 points to be distributed across this domain for the intra-domain weighing.		
DOMAIN D5. GLOBAL CHANGES.		
D5-1	Influence of rainfall on the abundance of vector populations.	
	Score 0	
	Score 1	Negligible: abundance of vector populations is not influenced by increased rainfall.
	Score 2	Low: abundance of vector populations is slightly influenced by increased rainfall.
	Score 3	Moderate: abundance of vector populations is moderately influenced by increased rainfall.
	Score 4	High: abundance of vector populations is highly influenced by increased rainfall.
D5-2	Influence of humidity on the survival and transmission of the pathogen/disease.	
	Score 0	
	Score 1	Negligible: abundance of vector populations is not influenced by increased humidity.
	Score 2	Low: abundance of vector populations is slightly influenced by increased humidity.
	Score 3	Moderate: abundance of vector populations is moderately influenced by increased humidity.
	Score 4	High: abundance of vector populations is highly influenced by increased humidity.
D5-3	Influence of temperature on the survival and transmission of the pathogen/disease.	
	Score 0	
	Score 1	Negligible: abundance of vector populations is not influenced by increased temperature.
	Score 2	Low: abundance of vector populations is slightly influenced by increased temperature.
	Score 3	Moderate: abundance of vector populations is moderately influenced by increased temperature.
	Score 4	High: abundance of vector populations is highly influenced by increased temperature.
D4-4	Changes in landscape, e.g., landscape fragmentation, creation of barriers, and landfill sites.	
	Score 0	
	Score 1	Negligible: changes in landscape have a negligible effect on the (re-)emergence/incidence of pathogen/disease.
	Score 2	Low: changes in landscape have a low effect on the (re-)emergence/incidence of the disease/pathogen but need other factors (e.g., land use changes combined with higher winter temperatures).
	Score 3	Moderate: landscape changes increases the density of reservoir hosts, availability of vectors, increases the pathogen’s survival, or increases the contact between humans and vectors. Furthermore, empty land can create a suitable environment for certain wildlife carrying the disease (e.g., migratory birds).
	Score 4	High: landscape changes are one of the main drivers for the pathogen, its vectors, or the contact between humans and vectors.
Number of drivers = 4, hence 40 points to be distributed across this domain for the intra-domain weighing.		
DOMAIN D6. WILDLIFE.		
D6-1	Potential roles of zoos in the (re-)emergence of the pathogen or increasing human disease incidence.	
	Score 0	
	Score 1	Negligible: the disease can be present in zoo animals but it is not known to have been transmitted from zoo animals to humans.
	Score 2	Low: the disease can enter a zoo (e.g., with introduction of an infected exotic animal) but only accidental transmissions of the disease from zoo animals to human have been reported. Hence, zoos have a low effect on the (re-)emergence of the disease or increasing human disease incidence in the country.
	Score 3	Moderate: the disease can enter a zoo and be present in zoo animals but it needs a vector (biological/mechanical) for its transmission to humans. Therefore, zoos have a moderate effect on the (re-)emergence of the disease or increasing human disease incidence in the country.
	Score 4	High: the disease can be introduced into a zoo via an infected imported animal; zoo animals can carry the disease that can be easily transmissed to humans.
D6-2	Increase of autochthonous (indigenous animal) wild mammals in Europe and neighbouring countries.	
	Score 0	Not applicable: the disease has not been reported in wildlife.
	Score 1	Negligible: the increase the autochthonous mammals population does not affect the risk of the human diseases to (re-)emerge.
	Score 2	Low: a slight increase of autochthonous mammals can slightly increase the probably of the human disease emerging/incidence.
	Score 3	Moderate: an increase of wild mammals has been associated with the re-emergence/incidence of the human disease.
	Score 4	High: an increase of wild mammals is the only factor associated with outbreaks of the disease in humans.
D6-3	Increase in endemic/migrating populations of wild birds.	
	Score 0	Not applicable: wild/migrating birds are not a reservoir of the disease or responsible for the spread of the pathogen through vector transport.
	Score 1	Negligible: there is a negligible probability of the disease (re-)emerging/increase of incidence in humans because of an increase in populations of endemic/migrating wild birds.
	Score 2	Low: there is a low probability of the disease (re-)emerging/increase of incidence and spreading through increased populations of endemic/migrating wild birds. Disease has spread from the endemic/migrating wild birds accidentally or under exceptional circumstances.
	Score 3	Moderate: there is a moderate probability of a disease being introduced and spread through increased populations of endemic/migrating wild birds.
	Score 4	High: there is a high probability for a disease to (re-)emerge/increase of incidence through increased populations of wild/migrating birds. These are hosts or reservoirs of the disease.
D6-4	Hunting Activities: hunted animals can be brought back to where livestock is present.	
	Score 0	
	Score 1	Negligible: the risk of the disease/pathogen (re-)emerging in livestock due to hunting activities is practically non-existent.
	Score 2	Low: disease is present in hunted wildlife and birds and only accidental cases have been reported in livestock that have (re-)emerged because of hunting. The risk of the disease/pathogen (re-)emerging in livestock due to hunting activities is practically non-existent.
	Score 3	Moderate: disease is present in hunted wildlife and birds but a certain control is established by the hunter.
	Score 4	High: disease is present in hunted wildlife and birds and hunting is one of the main modes of transmitting disease to livestock.
D6-5	Transboundary movements of terrestrial wildlife from other countries.	
	Score 0	Not applicable: pathogen or infected vector is not carried by terrestrial wildlife.
	Score 1	Negligible: (re-)emergence of disease or disease increasing by terrestrial movements of wildlife has only been suspected but never confirmed.
	Score 2	Low: there is a low probability for the disease to (re-)emerge and spread through transboundary movements of terrestrial wildlife.
	Score 3	Moderate: there is a moderate probability for the disease to (re-)emerge and spread through transboundary movements of terrestrial wildlife.
	Score 4	High: there is a high probability for the disease to (re-)emerge and spread through transboundary movements of terrestrial wildlife. These are host and may spread/carry the disease along.
Number of drivers = 5, hence 50 points to be distributed across this domain for the intra-domain weighing.		
DOMAIN D7. HUMAN ACTIVITIES.		
D7-1	Changes in human behaviour / activities leading to more contact with the pathogen in high risk areas (recreational activities, forest activities, mushroom picking, etc.)	
	Score 0	
	Score 1	Negligible: the changes of human behaviour are a negligible driver of the re-emergence/incidence of the disease in humans.
	Score 2	Low: the changes of human behaviour are a low driver of the re-emergence/incidence of the disease in humans.
	Score 3	Moderate: the changes of human behaviour are a moderate driver of the re-emergence/incidence of the disease in humans.
	Score 4	High: the changes of human behaviour are a high driver of the re-emergence/incidence of the disease in humans.
D7-2	Could changes in eating habits or consumer demands lead to an increased exposure to the hazard through foods?	
	Score 0	
	Score 1	Negligible: the changes in eating habits of consumers (e.g., demand for local and natural food such as raw milk cheese) are a negligible driver of the re-emergence/incidence of the disease.
	Score 2	Low: the changes in eating habits of consumers (e.g., demand for local and natural food such as raw milk cheese) are a low driver of the re-emergence/incidence of the disease.
	Score 3	Moderate: the changes in eating habits of consumers (e.g., demand for local and natural food such as raw milk cheese) are a moderate driver of the re-emergence/incidence of the disease.
	Score 4	High: the changes in eating habits of consumers (e.g., demand for local and natural food such as raw milk cheese) are a high driver of the re-emergence/incidence of the disease.
D7-3	People’s movements linked to tourism (infection of tourists during a visit to an infected country).	
	Score 0	
	Score 1	Negligible: tourism is a negligible driver on the emergence, re-emergence, or increasing human disease incidence in the country of interest.
	Score 2	Low: tourism increase has a low driver of the (re-)emergence or increasing human disease incidence in the country of interest.
	Score 3	Moderate: tourism increase has a moderate driver for the (re-)emergence or increasing human disease incidence in the country of interest. Biosecurity measures are enough to stop the entering of the pathogen.
	Score 4	High: tourist movement is a high driver on the (re-)emergence or increasing human disease incidence in the country of interest. Tourists are highly likely to bring the disease into your country in their belongings; biosecurity measures are insufficient to stop the pathogen.
D7-4	Human immigration.	
	Score 0	
	Score 1	Negligible: the immigration movements are a negligible driver of the disease (re-)emergence or increasing human disease incidence in the country of interest.
	Score 2	Low: the immigration movements are a low driver of the disease (re-)emergence or increasing human disease incidence in the country of interest.
	Score 3	Moderate: the immigration movement has a moderate effect as a driver on the (re-)emergence or increasing human disease incidence in the country of interest. Disease is highly likely to emerge using this route but biosecurity measures are enough to avoid (re-)emergence or increasing human disease incidence in the country of interest.
	Score 4	High: the immigration movement has a high effect as a driver on the (re-)emergence or increasing human disease incidence in the country of interest. Disease is highly likely to emerge using this route as biosecurity measures are not enough to avoid (re-)emergence or increasing human disease incidence in the country of interest.
D7-5	Transport movements: more specifically, commercial flights and commercial transport by ships, cars, or military (EXCLUDING TRANSPORT VEHICLES OF LIVE ANIMALS).	
	Score 0	
	Score 1	Negligible: the role of commercial movements as a driver on the (re-)emergence or increasing human disease incidence is negligible.
	Score 2	Low: the role of commercial movements as a driver on the (re-)emergence or increasing human disease incidence is low. It is easily preventable by implementing biosecurity measures.
	Score 3	Moderate: the role of commercial movements as a driver on the (re-)emergence or increasing human disease incidence is moderate. Disease can be prevented if biosecurity measures are tightened.
	Score 4	High: the role of commercial movements as a driver on the (re-)emergence or increasing human disease incidence is high. Disease is hard to control with the current biosecurity measures.
D7-6	Transport vehicles of live animals.	
	Score 0	
	Score 1	Negligible: the role of transport vehicles of live animals as a driver for the (re-)emergence or increasing human disease incidence is negligible.
	Score 2	Low: the role of transport vehicles of live animals as a driver for the (re-)emergence or increasing human disease incidence is low.
	Score 3	Moderate: the role of transport vehicles of live animals as a driver for (re-)emergence or increasing human disease incidence is moderate.
	Score 4	High: the role of transport vehicles of live animals as a driver for (re-)emergence or increasing human disease incidence is high.
D7-7	Bioterrorism potential.	
	Score 0	
	Score 1	Negligible: the role of bioterrorism as a driver for a disease to (re-)emerge is negligible: agent is available but difficult to handle or has a low potential of spread or generates few economic consequences.
	Score 2	Low: the role of bioterrorism as a driver for a disease to (re-)emerge is low: agent is available and easy to handle by professionals and labs but has a low spread.
	Score 3	Moderate: the role of bioterrorism as a driver for a disease to (re-)emerge is moderate: agent available and easy to handle by professionals, labs, and rapidly spreads.
	Score 4	High: the role of bioterrorism as a driver for a disease to (re-)emerge is high. Agent is available and easy to handle by individuals and rapidly spreads.
D7-8	Inadvertent release of an exotic infectious agent from a containment facility, e.g., laboratory.	
	Score 0	
	Score 1	Negligible: the pathogen is not currently present in any laboratory.
	Score 2	Low: the pathogen is present in a containment facility but its release is very unlikely as it is very easily contained.
	Score 3	Moderate: the pathogen is present in a containment facility and its release can occur as it is not easily contained.
	Score 4	High: pathogen is handled in a risk 3 or 4 laboratory (BSL3 or BSL4) in the country. It can leave the facility if the correct biosecurity measures are not implemented correctly and can easily spread to livestock.
Number of drivers = 8, hence 80 points to be distributed across this domain for the intra-domain weighing.		
DOMAIN D8. ECONOMIC AND TRADE ACTIVITIES.		
D8-1	Decrease of resources allocated to the disease surveillance in human and/or animal and/or environment.	
	Score 0	
	Score 1	Negligible: resources allocated to the disease surveillance have no effect on the (re-)emergence/incidence of the disease in your country. Disease has never been under surveillance.
	Score 2	Low: resources allocated to the disease surveillance have a low effect on the (re-)emergence/incidence of the disease in your country. Disease has been under surveillance in the past and no change has happened after surveillance has been stopped.
	Score 3	Medium: resources allocated to the disease surveillance have a moderate effect on the (re-)emergence/incidence of the disease in your country. Disease is under passive surveillance (reported only when observed) but with no need to further increase its surveillance
	Score 4	High: resources allocated to the disease surveillance have a high effect on the (re-)emergence of the disease in your country. Disease needs to be under active and passive surveillance as its (re-)emergence/incidence can easily occur. Therefore, if its surveillance decreases it is highly likely to (re-)emerge.
D8-2	Modification of the disease status (i.e., reportable disease becoming not reportable) or changes in screening frequency due to a reduced national budget.	
	Score 0	
	Score 1	Negligible: modification of the disease status due to a reduced national budget has a negligible effect on the (re-)emergence/incidence of the disease in your country.
	Score 2	Low: modification of the disease status due to a reduced national budget has a low effect on the (re-)emergence/incidence of the disease in your country.
	Score 3	Moderate: modification of the disease status due to a reduced national budget has a moderate effect on the (re-)emergence/incidence of the disease in your country.
	Score 4	High: modification of the disease status due to a reduced national budget has a high effect on the (re-)emergence/incidence of the disease in your country.
D8-3	Decrease of resources allocated to the implementation of biosecurity measures at border controls (e.g., harbors or airports).	
	Score 0	Endemic disease
	Score 1	Negligible: decreasing the resources allocated to the implementation of biosecurity measures has a negligible effect on the (re-)emergence or the human disease incidence in your country. Disease has never been detected in the past in a harbor or an airport.
	Score 2	Low: decreasing the resources allocated to the implementation of biosecurity measures has a low effect on the (re-)emergence or the human disease incidence in your country. The disease has been suspected to have entered other countries because of deficient biosecurity at border controls.
	Score 3	Medium: decreasing the resources allocated to the implementation of biosecurity measures has a moderate effect on the (re-)emergence or the human disease incidence in your country. The disease has been introduced in other countries because of deficient biosecurity at border controls.
	Score 4	High: decreasing the resources allocated to the implementation of biosecurity measures highly increases the risk of (re-)emergence or the human disease incidence in your country. In the past, the disease has been introduced in other countries AND in your country because of deficient biosecurity measures at border controls.
D8-4	Most likely influence of (il)legal movements of live animals (livestock, pets, horses, etc.) from neighbouring/European Union member states (MS) for the disease to (re-)emerge in your country.	
	Score 0	
	Score 1	Negligible: (il)legal movements of live animals (livestock, pets, horses, etc.) from neighbouring/European Union MS have a negligible influence on the pathogen/disease (re-)emergence/incidence in your country.
	Score 2	Low: (il)legal movements (livestock, pets, horses, etc.) from neighbouring/European Union MS have a low influence on the pathogen/disease (re-)emergence in your country.
	Score 3	Moderate: (il)legal movements (livestock, pets, horses, etc.) from neighbouring/European Union MS have a moderate influence on the pathogen/disease (re-)emergence in your country.
	Score 4	High: (il)legal movements (livestock, pets, horses, etc.) from neighbouring/European Union MS have a high influence on the pathogen/disease (re-)emergence in your country.
D8-5	Most likely influence of (il)legal EU trade exchanges of food products (e.g., raw milk cheese products) for disease (re-)emergence in your country.	
	Score 0	
	Score 1	Negligible: increased imports of dairy products such as raw milk cheese from European countries have a negligible influence on the pathogen/disease (re-)emergence in your country.
	Score 2	Low: increased imports of dairy products such as raw milk cheese from European countries have a low influence on the pathogen/disease (re-)emergence in your country.
	Score 3	Moderate: increased imports of dairy products such as raw milk cheese from European countries have a moderate influence on the pathogen/disease (re-)emergence in your country.
	Score 4	High: increased imports of dairy products such as raw milk cheese from European countries have a high influence on the pathogen/disease (re-)emergence in your country.
D8-6	Most likely influence of increased (il)legal imports of animal subproducts, such as skin, meat, and edible products from neighbouring/European Union member states (MS) for the disease to (re-)emerge in your country.	
	Score 0	
	Score 1	Negligible: Increased imports of animal subproducts, such as skin, meat, and edible products from neighbouring/European Union member states (MS) have a negligible influence on the pathogen/disease (re-)emergence in your country.
	Score 2	Low: Increased imports of animal subproducts, such as skin, meat, and edible products from neighbouring/European Union member states (MS) have a low influence on the pathogen/disease (re-)emergence in your country.
	Score 3	Moderate: Increased imports of animal subproducts, such as skin, meat, and edible products from neighbouring/European Union member states (MS) have a moderate influence on the pathogen/disease (re-)emergence in your country.
	Score 4	High: Increased imports of animal subproducts, such as skin, meat, and edible products from neighbouring/European Union member states (MS) have a high influence on the pathogen/disease (re-)emergence in your country.
D8-7	Most likely influence of increased (il)legal imports of NON-animal products, such as tires, wood, and furniture from EU member states for the disease/pathogen to (re-)emerge in your country.	
	Score 0	
	Score 1	Negligible: (il)legal movements of NON-animal products, such as tires, wood, and furniture from EU member states for the disease/pathogen to (re-)emerge in your country have a negligible influence on the pathogen/disease (re-)emergence/incidence in your country.
	Score 2	Low: (il)legal movements of NON-animal products, such as tires, wood, and furniture from EU member states for the disease/pathogen to (re-)emerge in your country have a low influence on the pathogen/disease (re-)emergence/incidence in your country.
	Score 3	Moderate: (il)legal movements of NON-animal products, such as tires, wood, and furniture from EU member states for the disease/pathogen to (re-)emerge in your country have a moderate influence on the pathogen/disease (re-)emergence/incidence in your country.
	Score 4	High: (il)legal movements of NON-animal products, such as tires, wood, and furniture from EU member states for the disease/pathogen to (re-)emerge in your country have a high influence on the pathogen/disease (re-)emergence/incidence in your country.
D8-8	Most likely influence of (il)legal movements of live animals (livestock, pets, horses, etc.) from third-world countries for the disease to (re-)emerge in your country.	
	Score 0	
	Score 1	Negligible:(il)legal movements of live animals (livestock, pets, horses, etc.) from third-world countries have a negligible influence on the pathogen/disease (re-)emergence/incidence in your country.
	Score 2	Low: (il)legal movements of live animals (livestock, pets, horses, etc.) from third-world countries have a low influence on the pathogen/disease (re-)emergence/incidence in your country.
	Score 3	Moderate: (il)legal movements of live animals (livestock, pets, horses, etc.) from third-world countries have a moderate on the pathogen/disease (re-)emergence/incidence in your country.
	Score 4	High: (il)legal movements of live animals (livestock, pets, horses, etc.) from third-world countries have a high influence on the pathogen/disease (re-)emergence in your country.
D8-9	Most likely influence of (il)legal third-world countries trade exchanges of food products (dairy raw products) for disease (re-)emergence in your country.	
	Score 0	
	Score 1	Negligible: increased imports of dairy products such as raw milk cheese from third-world countries have a negligible influence on the pathogen/disease (re-)emergence/incidence in your country.
	Score 2	Low: increased imports of dairy products such as raw milk cheese from third-world countries have a low influence on the pathogen/disease (re-)emergence/incidence in your country.
	Score 3	Moderate: increased imports of dairy products such as raw milk cheese from third-world countries have a influence on the pathogen/disease (re-)emergence/incidence in your country.
	Score 4	High: increased imports of dairy products such as raw milk cheese from third-world countries have a high influence on the pathogen/disease (re-)emergence/incidence in your country.
D8-10	Most likely influence of increased imports of animal subproducts, such as skin, meat, and edible products from third-world countries, for the disease to (re-)emerge in your country.	
	Score 0	
	Score 1	Negligible: increased imports of animal subproducts, such as skin, meat, and edible products from third-world countries have a negligible influence on the pathogen/disease (re-)emergence/incidence in your country.
	Score 2	Low: increased imports of animal subproducts, such as skin, meat, and edible products from third-world countries have a low influence on the pathogen/disease (re-)emergence/incidence in your country.
	Score 3	Moderate: increased imports of animal subproducts, such as skin, meat, and edible products from third-world countries have a moderate on the pathogen/disease (re-)emergence/incidence in your country.
	Score 4	High: increased imports of animal subproducts, such as skin, meat, and edible products from third-world countries have a high influence on the pathogen/disease (re-)emergence/incidence in your country.
D8-11	Most likely influence of increased (il)legal imports of NON-animal products, such as tires, wood, and furniture from third-world countries, for the disease to (re-)emerge in your country.	
	Score 0	
	Score 1	Negligible: increased (il)legal imports of NON-animal products, such as tires, wood, and furniture from third-world countries have a negligible influence on the pathogen/disease (re-)emergence/incidence in your country.
	Score 2	Low: increased (il)legal imports of NON-animal products, such as tires, wood, and furniture from third-world countries have a low influence on the pathogen/disease (re-)emergence/incidence in your country.
	Score 3	Moderate: increased (il)legal imports of NON-animal products, such as tires, wood, and furniture from third-world countries have a moderate influence on the pathogen/disease (re-)emergence/incidence in your country.
	Score 4	High: increased (il)legal imports of NON-animal products, such as tires, wood, and furniture from third-world countries have a high influence on the pathogen/disease (re-)emergence/incidence in your country.
Number of drivers = 11, hence 110 points to be distributed across this domain for the intra-domain weighing.		

**Table A2:** Profiles of Experts Involved in the Elicitation of Knowledge, in alphabetic order (N = 40).

ID	Name	Surname	Gender	ISO	Level	Employment	Competency	Keyword 1	Keyword 2	Keyword 3
1.	Berger	Thomas F.H.	M	CH	N; R; I	GI; RS	FS	Dairy	Food safety	Risk evaluation and mitigation
2.	Bødker	René	M	DK	N	UN	PH; AH	VBD	Epidemiology	Surveiilance
3.	Bonnet	Sarah I.	F	FR	N; R; I	RS	PHAHEH	Ticks	TBP	Tick-host-pathogen interactions
4.	Boulanger	Nathalie	F	FR	N; I	GI; UN; RS	PH; LD	Medical entomologist	Parasitologist	Infectious diseases
5.	Bournez	Laure	F	FR	N	RS	AH; EH	Eco-Epidemiology	Ticks and TBP	TBEV
6.	Brugger	Katharina	F	AT	NI	RS	PH; EH	Climatology	Epidemiology	Vectors and VBD
7.	De Regge	Nick	M	BE	N	GI	AH; LD	Virology	VBD	Diagnostics
8.	Estrada-Peña	Agustín	M	SP	N	UN	PH; AH	Ticks	Ecology, Epidemiology, and Vector hosts	Zoonoses
9.	Fravalo	Philippe	M	FR	N	UN	PH; FS; LD	Biological risk in food	Meat production chain	Pathogen characterization
10.	Geller	Julia	F	EE	N	GI; RS	PH	Research	Monitoring	Surveillance
11.	Gilot-Fromont	Emmanuelle	F	FR	N	UN	AH; EH	Eco-epidemiology	Disease management	
12.	Gonzalez	Gaëlle	F	FR	N	GI	AH	Surveillance	Virulence determinants	Co-infection
13.	Haddad	Nadia	F	FR	N	UN; RS	PH; AH	Zoonoses	TBP	Epidemiology
14.	Heyman	Paul	M	BE	N; I	GI; DL	PH; AH; LD	TBD	Hantaviruses	
15.	Hoch	Thierry	M	FR	N	RS	AH	Matematical modelling	Epidemiology	Ticks
16.	Humblet	Marie-France	F	BE	R	UN	AH	Animal biosecurity	Veterinary epidemiology	One Health
17.	Jourdain	Elsa	F	FR	N	GI	PH; AH; LD	Descriptive epidemiology	Zoonotic diseases	Animal reservoir
18.	Kerlik	Jana	F	SK	R	GI	PH	Epidemiology	Zoonoses	TBE
19.	Klein	Matthias	M	DE	R	UN	PH	Neuroinfectiology	Emergency medicine	Intensive care
20.	Knap	Natasa	F	SI	N	UN; RS; DL	LD	Diagnostics	Ecology	Virology
21.	Kooh	Pauline	F	FR	N	GI	FS	Food Microbiology	Risk assessment	Virology
22.	Lacour	Sandrine A.	F	FR	N	GI	AH; FS	Molecular virology	Virus-host interactions	Milk/cheese-borne TBEV
23.	Leandri	Marc	M	FR	N	UN	EC	Decision	Perceptions	Information
24.	Leib	Stephen L.	M	CH	N; R; I	UN; RS; DL	LD	Infectious Diseases	Microbiology	Brain infections
25.	Lernout	Tinne	F	BE	N	RS	PH	TBD	Epidemiology	Risk Assessement
26.	L’Hostis	Monique	F	FR	N	UN	PH	Parasitologist	*Ixodes ricinus*	Ecology
27.	Martin-Latil	Sandra	F	FR	N	GI	FS; LD	Virus	Detection	Water / food
28.	Moutailler	Sara	F	FR	N	GI; RS	AH	Ticks and TBP	Vector competence	Molecular epidemiology
29.	Purse	Bethan	F	UK	I	RS	PH; AH; EH	VBD	Ecology	Epidemiological modeling
30.	Quillery	Elsa	F	FR	N	GI	PH	Ecology	Vector diseases	Risk assessment
31.	Raffetin	Alice	F	FR	N; R; I	UN; RS; HO	PH	Lyme borreliosis	TBD	Infectious diseases
32.	Ruiz-Fons	Francisco	M	SP	N; R; I	GI; RS	AH	Epidemiology	Wildlife	VBP
33.	Ruzek	Daniel	M	CZ	N	UN	AH; LD	TBE	Flaviviruses	Virus-host interactions
34.	Solveig	Jore	F	NO	N	RS	PH	Zoonoses	TBD	Climate change
35.	Sprong	Hein	M	NL	NI	GI; UN	PH; EH; LD	TBD	Molecular epidemiology	Disease ecology
36.	Studahl	Marie	F	SE	N; R	UN; RS; HO	PH	Viral CNS infections	Viral diagnostics	Burden of infectious diseases
37.	Thiry	Etienne	M	BE	N	UN	AH; LD	Animal viruses	Veterinary infectiology	Veterinary Public Health
38.	Velay	Aurélie	F	FR	R	UN; RS; DL	PH; LD	Virology	Emerging Diseases	TBE
39.	Zdenek	Hubalek	M	CZ	N; I	RS	PH; AH; EH	Arbovirology	Zoology	Monitoring
40.	Zomer	Tizza	F	NL	R	HO	PH	Lyme borreliosis	Epidemiology	TBD

Legend: ISO, the standard defining codes for the names of countries; Keywords: maximum three and in decreasing order of importance; TBE, tick-borne encephalitis; TBEV, tick-borne encephalitis virus; TBD, tick-borne diseases; TBP, tick-borne pathogens; VBD, vector-borne diseases. Level: N, national; R, regional; I, international. Employment: GI, government institution; UN, university; RS, research/scientific institution; DL, diagnostic laboratory; HO, hospital. Competency: PH, public health; AH, animal health; EH, environmental health; FS, food safety; LD, laboratory diagnostic; EC, economics.

## Appendix B. Guidance Letter for the Expert Elicitation

Ranking criteria of the tick-borne encephalitis disease with an observed emergence and an increasing incidence in humans across Europe (expert opinion)

___________________________________________________________________

Dear experts and colleague,

This special request is related to the (re-)emergence or increasing incidence of tick-borne encephalitis disease in humans across Europe.

The objective of the present study is to better understand the drivers (i.e., influencing factors) of the (re-)emergence or increase in incidence of the tick-borne encephalitis disease in humans across Europe. The questionnaire was prepared in order to present different criteria of interest and summarized through 59 drivers. For each driver, scores are proposed with a corresponding definition. Drivers are grouped per category (N = 8), with each category corresponding to one spreadsheet. After scoring, the weight of each driver in a specific category and in each category is calculated.

Objectives of the questionnaire

We would like your expert’s opinion on the drivers of the (re-)emergence or increasing incidence of tick-borne encephalitis disease in humans across Europe.

Hence, to answer on the basis of “how likely is it for tick-borne encephalitis disease to (re-)emerge or increase in incidence in humans across Europe in response to the different drivers?”.

How to fill out the questionnaire

In the attached Excel questionnaire, there are 11 spreadsheets. The first one summarizes your expertise. The next eight correspond to the eight categories of drivers. The tenth spreadsheet corresponds to the intra-category weighing, and the last one to the level of uncertainty per category of drivers.

1. Expert information
2. Pathogen characteristics: 12 criteria
3. Distance to Europe and your country: 3 criteria
4. Ability to monitor, treat, and control the disease: 11 criteria
5. Farm/European characteristics: 5 criteria
6. Global change: 4 criteria
7. Wildlife interface: 5 criteria
8. Human activities: 8 criteria
9. Economy and trade activities: 11 criteria
10. Intra-category weighing
11. Level of uncertainty per domain of criteria

Actions to be performed:

(1) Score and balance each driver within each category of drivers:
  ▪ Please give a score according to what you estimate is the importance of each driver in the (re-)emergence or increasing incidence of tick-borne encephalitis disease in humans across Europe.
  ▪ After scoring, please balance each driver for each category of drivers. Balancing the criteria will depend on the distribution of points among the different criteria in each category. The total number of points to be distributed among the drivers is specified for each category (on each spreadsheet). For example, in the category “pathogen characteristics”: a total of 120 points must be distributed => for “distance to Europe and your country”, a total of 30 points must be distributed. If a driver has no importance related to others, zero points can be attributed to this driver.
(2) Intra-category weighing:

The next step of the process will consist of the distribution of 100 points among the 8 categories of criteria (pathogen characteristics, distance of outbreaks, etc.). This is on the 10th spreadsheet. The distribution will depend on which is believed to be the strongest category of drivers.

(3) Level of uncertainty:

In the last spreadsheet, give an overall level of uncertainty for each category of characteristics using a scale from 0 (minimal uncertainty in my scoring) to 100 (maximum uncertainty in my scoring).

As an expert in the field, your collaboration is essential and will help us a lot in the successful completion of this project.

Thank you in advance for your collaboration and for the time spent filling out the file before September 30, 2022.

Each expert who contributes to the study (i.e., questionnaire fully completed in due time) will be added as a co-author in the expected publication with the results of the elicitation.

For any question, please contact claude.saegerman@uliege.be

Kind Regards,

Professor Claude Saegerman

Research Unit in Epidemiology and Risk

Analysis applied to Veterinary Sciences

Fundamental and Applied Research for Animal and Health

Department of Infectious and Parasitic Diseases

Faculty of Veterinary Medicine

University of Liège

Quartier Vallée 2

Avenue de Cureghem 7A

4000 Liège Sart-Tilman

BELGIUM

Tél.: +32 (0)4 366 45 79

E-mail: claude.saegerman@uliege.be

**Table A3:** Grouping of Countries of Origin of the Elicited Experts based on Level of Endemicity Regarding the Tick-Borne Encephalitis.

Country of Origin	Number of Cases 2020	Incidence 2020	Short Description of the Situation	Trend in Number of Cases 2012−2020 (Country Level)	Proposed Level (Increasing Order: A to E)
Spain	0	0	No autochthonous cases reported.	-	A
France	46	0.1	Low endemicity, only in few regions	Increase trend	B
Belgium	7	0.1	Sporadic cases (emergence in 2020)	-	
The Netherlands	5		Sporadic cases (emergence in 2016)	-	
Denmark			Sporadic cases (endemic only on Bornholm island)	No information	
United Kingdom	2	0	Sporadic cases (emergence in 2019)	-	
Norway	41	0.8	Low to moderate endemicity, only in few regions	Increase trend	C
Austria	250	2.8	Low to moderate endemicity, throughout the country	Increase trend	D
Germany	705	0.5	Low to high endemicity, throughout the country	Increase trend	
Sweden	267	2.6	Low to high endemicity, in most of the country	Increase trend	
Slovakia	185	3.4	Low to high endemicity, throughout the country	No trend	
Switzerland	454	5.1	Moderate to high endemicity, in most of the country	Increase trend	
Czechia	849	7.9	Moderate to high endemicity, throughout the country	Increase trend	E
Estonia	70	5.3	Moderate to high endemicity, throughout the country	Decrease trend	
Slovenia	187	8.9	Low to high endemicity, throughout the country	No trend	
